# Shift in diet composition of a riparian predator along a stream pollution gradient

**DOI:** 10.1098/rspb.2024.2104

**Published:** 2024-11-20

**Authors:** Maike Huszarik, Alexis P. Roodt, Teagan Wernicke, Moritz Link, Eva Lima-Fernandes, David Åhlén, Verena C. Schreiner, Ralf Schulz, Peter Hambäck, Martin H. Entling

**Affiliations:** ^1^ iES Landau, Institute for Environmental Sciences, University of Kaiserslautern-Landau (RPTU), Fortstraße 7, Landau in der Pfalz 76829, Germany; ^2^ Field Station Fabrikschleichach, Chair of Conservation Biology and Forest Ecology, Biocenter, University of Würzburg, Glashüttenstraße 5, Rauhenebrach 96181, Germany; ^3^ Department of Ecology, Environment and Plant Sciences, Stockholm University, Stockholm 10691, Sweden; ^4^ Faculty of Biology, University of Duisburg-Essen, Universitätsstrasse 2, Essen 45141, Germany; ^5^ Research Center One Health Ruhr, University Alliance Ruhr, Universitätsstrasse 2, Essen 45141, Germany; ^6^ Eußerthal Ecosystem Research Station, University of Kaiserslautern-Landau (RPTU), Birkenthalstraße 13, Eußerthal 76857, Germany

**Keywords:** molecular gut content analysis, trophic interactions, pharmaceuticals, pesticide toxicity, riparian forest, Araneae

## Abstract

Terrestrial insectivores in riparian areas, such as spiders, can depend on emergent aquatic insects as high-quality prey. However, chemical pollution entering streams from agricultural and urban sources can alter the dynamics and composition of aquatic insect emergence, which may also affect the riparian food web. Few studies have examined the effects of stressor-induced alterations in aquatic insect emergence on spiders, especially in terms of chemical pollution and diet composition. We used DNA metabarcoding of gut content to describe the diet of *Tetragnatha montana* spiders collected from 10 forested streams with differing levels of pesticide and wastewater pollution. We found that spiders consumed more *Chironomidae* and fewer other aquatic Diptera, including Tipulidae, Ptychopteridae and Culicidae, at more polluted streams. Pollution-related effects were mainly observed in the spider diet, and were not significant for the number nor composition of flying insects trapped at each site. Our results indicate that the composition of riparian spider diets is sensitive to stream pollution, even in the absence of a change in the overall proportion of aquatic prey consumed. A high reliance on aquatic prey at polluted streams may give spiders an increased risk of dietary exposure to chemical pollutants retained by emergent insects.

## 1. Introduction

Terrestrial and aquatic ecosystems are closely linked by a transfer of nutrients across the land–water interface, which can act as subsidies to the recipient ecosystem [1]. Aquatic subsidies—namely emergent insects with an aquatic larval phase—represent high-quality prey for terrestrial predators (e.g. spiders [2]; birds [3]; bats [4]). They are rich in essential long-chain polyunsaturated fatty acids that are not commonly found in terrestrial insect prey [5,6]. Owing to their important role in the riparian food web, changes in emergent aquatic insect abundance, phenology or quality can have negative consequences for terrestrial insectivores [7–9].

One group of consumers of emergent aquatic insects (hereafter emergent insects) are spiders living in riparian areas. Orb weavers such as *Tetragnatha* spp. can be highly reliant on emergent insect prey [10–12], and their distribution along streams is influenced by the presence of emergent insects [13,14]. Spiders link terrestrial and aquatic food webs, as they also serve as prey for other predators such as birds and bats [15]. Furthermore, shifts in riparian spider diet owing to emergent insect subsidies may alter top-down effects in the terrestrial food web [16,17].

Stressors in streams can indirectly affect riparian insectivores by altering insect emergence [18,19]. Depending on their properties, chemical pollutants such as pesticides and pharmaceuticals can directly affect insects in the stream ecosystem [20,21]. Several field studies have observed negative relationships between stream pollution and riparian insectivores (such as birds: [22] and bats: [23]), although many aspects, including indirect effects through specific dietary changes, remain unresolved. In terms of spiders, stream pollution has been associated with a reduction in the abundance and diversity of riparian spiders [8,24]. Moreover, the contribution of aquatic prey to the diet of riparian spiders changed along an agricultural pollution gradient, though this change was specific to different spider species [25]. However, little is known about how stream pollution affects spider diets beyond changes in the overall proportions of aquatic and terrestrial prey consumed, especially with regards to changes in the specific prey taxa.

Many of the studies investigating effects of aquatic insect emergence on insectivore diets thus far have used stable isotope analysis (SIA), including for spiders (e.g. [12,25]). SIA is an effective method for determining main sources, contribution and types of prey consumed, but it is less suitable for identifying specific taxa (i.e. family or species) responsible for changes, or lack thereof, in aquatic diet contribution [26]. Molecular gut content analysis using DNA metabarcoding to detect prey DNA is a highly sensitive method that is ideal for detecting species-level changes in spider diets ([27,28]; for limitations, see [29]). This high-resolution molecular approach can complement information already obtained by SIA (as in [30]) and could be used to improve our understanding of how stream pollution affects trophic links in riparian food webs at a detailed level.

The aim of our study was to describe changes in the diet of riparian spiders along a stream pollution gradient. This is the first study, to our knowledge, that uses DNA metabarcoding of spider gut contents to investigate indirect trophic effects of chemical stream pollution on riparian spiders. Stream pollution in terms of pesticides and wastewater markers was quantified at stream sites surrounded by riparian forests in a parallel study [23]. In the present study, we analyse *Tetragnatha montana* specimens that were collected from the same sites, as they are common along streams and are known to have a high, but variable, proportion of aquatic prey in their diet [12,25]. We also captured and identified the potential flying insect prey at each stream to characterize their relationship with the spider diet. We expected that the proportion of aquatic prey detected in the spider diet would reflect the abundance and proportion of flying emergent insects sampled at the streams. Furthermore, previous studies show that the overall abundance of emergent insects may not necessarily decrease in polluted streams, as sensitive species are replaced by tolerant ones [31,32]. Based on this and the results from our parallel study [23], we expected that Diptera tolerant to stream pollution, such as Chironomidae [33], would be more common in the diet of spiders collected from more polluted streams, signifying a change in their diet composition in response to stream pollution.

## 2. Methods

### (a) Field study sites

The field study was conducted at 10 stream sites located in riparian forest areas in Rhineland-Palatinate, Germany (electronic supplementary material, table S1, figure S1). The stream sites were part of a larger field study evaluating effects of chemical stream pollution on riparian bats [23]. Streams in this area flow from the Palatinate Forest, a UNESCO Biosphere Reserve, to the Rhine River, passing through viticulture, other agriculture, urban and forest land uses. Each site consisted of a 40 m stretch of stream with a relatively natural structure (no more than ‘moderately altered’; [34]), surrounded by predominantly deciduous forest and away from direct exposure to agricultural or urban land use. However, the sites differed in their amounts of upstream agricultural and urban areas (pollution sources). The field study was conducted from 21 April to 1 July 2020, during the region's main pesticide application period, when the highest loads and effects of pesticide pollution are expected [35].

Sites were visited once per week to measure physicochemical stream characteristics and collect water samples for pollutant analysis. The stream width and depth were measured, the concentrations of dissolved nutrients were measured with a nutrient analysis kit (VISOCOLOR® ECO reagents with PF-12 Spectrophotometer; Macherey-Nagel GmbH, Germany), and a multi-parameter meter (Multi 3620 IDS or Multi 340i, WTW Xylem Analytics GmbH, Germany) was used to measure the water temperature, pH, dissolved oxygen and conductivity. Tree canopy cover over the streams, vegetation surface clutter (i.e. any vegetation on the stream surface) and the separation between shrubs along the shore were characterized on 23 June 2020 ([36]; electronic supplementary material, tables S2 and S3). Vegetation, such as tree canopy cover or vegetation emerging from the water, can influence the production of emergent insects in streams [37,38] and *Tetragnatha* spp. diet [14]. All stream variables are presented in electronic supplementary material, table S4.

### (b) Analysis of chemical pollutants in stream water

Weekly water grab samples were collected mid-stream to measure pesticide and wastewater pollutants, using 1 l amber glass bottles. To capture peak contamination levels that may be missed by grab sampling [39], high-water-level event samples were collected during a single rain event occurring at all sites using 1 l bottle samplers (electronic supplementary material, figure S2). Chemical pollutants were then extracted from the stream water by solid phase extraction (SPE), following Machado *et al*. [40], using Oasis® HLB 6 cc 500 mg SPE cartridges (Waters Corporation, Milford, USA). High-performance liquid chromatography tandem to triple quadrupole mass spectrometry by electrospray ionization (HPLC-ESI-MS/MS) was used to detect and measure the concentrations of 77 pesticides and 4 established wastewater markers in the extracted water samples (electronic supplementary material, table S5) using an Agilent 1260 Infinity II HPLC system tandem to an Agilent 6495 triple quadrupole mass spectrometer (MS/MS; Agilent Technologies, Inc., Santa Clara, CA, USA). Further details of the extraction, chemical analysis, processing and quality assurance procedures are described in Huszarik *et al*. [23].

The potential cumulative pesticide toxicity of each stream sample was calculated as the logarithmic sum toxic unit (*sumTU*; [41]) for freshwater invertebrates:


(2.1) $$sumTU=\log_{10}\left(\frac{C_{i}}{EC_{50_{i}}}\right)$$

where $C_{i}$ is the normalized concentration of pesticide i and ${EC}_{50_{i}}$ is the concentration affecting 50% (EC_50_) of organisms in an acute test with pesticide i. Acute exposure (24–96 h) EC_50_ values for the most sensitive freshwater invertebrate were mainly obtained from the ECOTOX database [42] using the R package Standartox [43], or the PPDB [44]. Wastewater markers indicating treated (carbamazepine, diclofenac and sulfamethoxazole) and untreated (caffeine) wastewater [45,46] were only evaluated qualitatively, as concentrations of wastewater effluent in streams can change hourly [47] and sites were visited at various times of day. We totalled all detections of wastewater markers for each stream site over the study period. Stream pollution variables are presented in electronic supplementary material, table S4.

### (c) Collection of potential flying insect prey

We sampled flying insects at the stream shores continuously for one week on four separate occasions (collection dates: 12/13 May, 19 May, 2 June, 9/10 June 2020) using ‘Sea, Land, Air’-style Malaise (SLAM) traps (MegaView Science Co., Ltd., Taichung, Taiwan). The Malaise traps were set 1 m above the water surface, directly above the stream shoreline with openings parallel to the stream (electronic supplementary material, figure S3). The collection bottles were filled with propylene glycol trapping medium (33% propane-1,2-diol, 66% water, 1 ml l^–1^ dish soap and 10 mg l^–1^ denatonium benzoate for deterring larger animals). Samples were collected from the bottles after one week. Flying insects were counted and identified to order or to family level for orders that include families with both aquatic and terrestrial larval origin (i.e. Diptera and Coleoptera; electronic supplementary material, table S6; [48,49]).

### (d) Spider collection

Between 11 and 29 adult *Tetragnatha montana* were collected at each stream site from 29 May to 4 July 2020, and subsequently used for diet analysis (196 individuals total; electronic supplementary material, table S7). Spiders were sampled in areas immediately downstream of the field sites (average within 88 m of the site, up to 200 m downstream, spiders sampled 75 m upstream at one site; electronic supplementary material, table S7); this avoided disturbing the streambed where other sampling was taking place while ensuring that the same habitat and pollution qualities were maintained [23]. Spiders were collected individually from their webs directly above the water surface, when possible, or along the stream shore (one spider, within 5 m of the stream) using sterile tweezers. They were stored individually in PVC vials and then frozen at −18°C for one night. On the following day, they were transferred to 98% denatured EtOH and stored at −20°C before further processing.

### (e) DNA extraction and amplification

Spiders were first prepared under sterile conditions to reduce the amount of spider material included in the DNA extraction of gut contents. Complete dissection of the stomach is difficult to perform on spiders owing to their complex digestive system [50]. Instead, spiders were dissected on sterile filter paper as follows: once excess ethanol had evaporated, the legs and pedipalps or epigynes were removed and the spider was carefully cut in half lengthwise. One half of the abdomen and cephalothorax was used for DNA extraction. If the abdomen was small (abdomen length <4 mm) then the entire abdomen and cephalothorax were used, without legs. The dissected spider parts were stored dry at −20°C until DNA extraction.

DNA extraction was performed semi-automatically using the Mag-Bind® Blood & Tissue DNA HDQ 96 kit (Omega Bio-Tek, Inc., Norcross, GA, USA) containing magnetic beads, following a modified protocol (guide for tissue, OBT_M6399_Kduo_100 µL_v1.1; electronic supplementary material, section S1). Briefly, each spider sample was homogenized, then incubated overnight in a tissue-lysis buffer and proteinase K solution. The next day, 5 µl of 10 mg ml^–1^ RNAse were added and samples were incubated at room temperature, then centrifuged (10 min at 40 000×g; CT15RE, VWR Hitachi, Lutterworth, UK). Buffers were added to the extraction plate wells as described in the protocol, followed by 250 µl of the sample supernatant, binding buffers and magnetic beads. Automated extraction was performed with the KingFischer™ Duo Prime Purification System (Thermo Fischer Scientific, Inc., Waltham, MA, USA). Following extraction, the concentrations of DNA per sample were measured with the NanoDrop™ One (Thermo Fischer Scientific, Inc., Waltham, MA, USA). Samples were then stored at −20°C. A blank extraction sample was included with each extraction day to control for DNA contamination (*n* = 3).

Prey DNA was amplified using tagged NoAranR (reverse primer, 5′−3′ TGTTCATCCDGTNCCWG; [27]) and LCO1490 (forward primer, 5′−3′ GGTCAACAAATCATAAAGATATTGG; [51]) primers in a single polymerase chain reaction (PCR) step (‘tagged PCR protocol’; [52]). Both forward and reverse primers included 8 base-pair tags (electronic supplementary material, table S8; [53]). This primer combination is designed to preferentially amplify insect DNA while reducing amplification of spider DNA [27]. It should be noted that the amplification reduction by these primers is poorer for *Tetragnatha* spp. than for other spiders [27]. Ten microlitres of each primer (10 µM; 0.4 µM in PCR mix), 25 µl of Multiplex PCR Master Mix (QIAGEN GmbH, Germany) and 5 µl of extracted DNA (concentration: 20–30 ng µl^–1^, 2–3 ng µl^–1^ in PCR mix) were combined under sterile conditions. Each sample within a PCR run (up to 64 samples) received a unique tagged primer combination. Any samples with DNA concentrations over 40 ng µl^–1^ were diluted with sterile ultrapure water before being added to the PCR reagents. For PCR blanks, 5 µl of sterile ultrapure water were added instead of DNA. Approximately 25% of the primer combinations per plate were blanks to monitor the frequency of sequencing errors [52]. Details of the PCR protocol are provided in the electronic supplementary material, table S9. The amplified samples were stored at 4°C and success was verified using gel electrophoresis. The concentration of amplified DNA was measured using a Qubit v.2.0 Fluorometer with the Qubit dsDNA HS Assay Kit (Thermo Fischer Scientific, Inc., Waltham, MA, USA).

Following amplification, samples were combined into seven pools (one pool per PCR plate), adjusting the volume based on the amplified DNA concentrations to ensure that each sample contributed a similar amount of DNA. Pools were cleaned using AMPure XP beads, following the manufacturer’s protocol (1.8 times the pool volume; Beckman Coulter, Brea, CA, USA), to remove excess nucleotides and primers. The concentration of DNA was re-measured with the Qubit HS kit. Pools were then cleaned again using the MinElute PCR Purification kit (QIAGEN GmbH, Germany). A unique combination of forward and reverse Illumina TruSeq® DNA Single Index adaptors (Set A: i2, i4, i5, i6, i7, i13, i19; Illumina, Inc., San Diego, CA, USA) were then added to each pool using a phosphorylation and adapter ligation step (electronic supplementary material, §S2). Finally, the pools were purified using the MinElute Gel extraction Kit (QIAGEN GmbH, Germany), targeting a fragment length of 300 bp. Pools were then concentrated, pooled into a final library and sequenced by the SNP&SEQ Technology Platform at the Science for Life Laboratory in Uppsala Sweden using a MiSeq™ system and v3 sequencing chemistry (Illumina, Inc., San Diego, CA, USA).

### (f) Bioinformatics

Output sequences were processed using ObiTools [54] in the galaxy web interface (use.galaxy.eu, 2023; [55]). Paired-end sequences of high quality (score >40) were assembled using the tool ‘Illuminapairedend’ and demultiplexed with the tool ‘NGSfilter’ after filtering for size. We then used the tool ‘obiuniq’ to identify and count unique sequences before clustering operational taxonomic units (OTUs) using a 97% similarity threshold, tabulated for each spider individual.

Taxonomic assignments were matched to OTU sequences using BOLD (accessed 16.05.2023; [56]) within the BOLDigger interface (v. 2.1.2; [57]). Sequences were aligned using the BOLDigger pipeline to find the top 20 matches, including the ‘Correction of top hits via BOLD API’ option, using a threshold of 97% similarity for taxonomic assignment. Assignments were made to the most specific taxonomic level of the best match. Multiple matches with the same similarity, private or early release sequence matches, or with suspicious matches were confirmed by BLASTing in GenBank using Megablast (BLASTN v. 2.13; [58]) to obtain the top 30 matches. Any better match, based on identity score, match length and E-value, was selected. We verified the geographic range of taxonomic matches using the Global Biodiversity Information Facility (https://gbif.org, accessed May 2023); only species with occurrence records in Germany or neighbouring countries were included. Finally, any OTUs with only one read in a single sample or blank (singletons) were removed from the OTU table. All non-arthropod OTUs were removed, as well as *Tetragnatha* spp., to retain only arthropod prey OTUs.

We used read count thresholds to filter OTUs as being present (detected) or absent in spider samples. We did not find evidence for DNA contamination in blank samples. Therefore, we set a minimum read count threshold of 0.1% of the total OTU read count, above which an individual OTU was considered as detected in a sample. This threshold is designed to limit the occurrence of false-positives or artefacts in the sequencing results [59]. If the maximum number of reads in a blank was higher than the threshold, which only occurred for five OTUs, then the maximum read count in the blanks was used as a threshold to exclude any erroneous detections [60].

Following presence/absence filtering, we added together the number of detections of OTUs sharing identical taxonomic names (e.g. same species) to obtain the total number of detections of each prey taxon per spider. We also grouped all prey taxa in higher taxonomic groups (family or order), based on their terrestrial or aquatic larval origin (electronic supplementary material, table S6) and added all detections within each taxonomic group together for each spider. Finally, the proportions of detections belonging to each taxa group or taxon, as well as the proportions of detections belonging to aquatic and terrestrial prey, were calculated for each stream site. This was done by pooling the detections of all spiders at each site, then dividing the number of detections of each specified group or taxon by the total number of detections at that site (see electronic supplementary material, table S10).

### (g) Data analysis

Weekly measurements for each physicochemical variable and the sumTU were averaged per stream site over the study period. Highly correlated variables were excluded from statistical analyses. To identify environmental gradients present across the stream sites and relationships between measured variables, we conducted a principal component analysis (PCA; vegan [61]) of variables describing physical stream characteristics and in-stream chemical pollution (electronic supplementary material, table S4). For the statistical analyses, we selected three variables *a priori* based on their importance for insect emergence and riparian spiders, and their representation of environmental gradients observed at the sites. The potential pesticide toxicity of the streams (average sumTU) is directly related to negative effects of pesticides on emergent insects and indirect effects on spider diet [25,62]. In addition, stream width and tree canopy cover are both relevant for insect emergence and spiders, which can be affected by the size and vegetation coverage of streams [14,37,38,63].

Thus, we assessed the relationship between pesticide toxicity, stream width and canopy cover and the composition of both the flying insect community and *T. montana* diet using permutational multivariateanalysis of variance (PERMANOVA with ‘adonis’; vegan [61]). In addition to the higher classification (order to family level) of the prey taxonomic groups, the spider diet composition was also tested at the species level by including all detected prey taxa (electronic supplementary material, table S10). Permuted linear models were used to determine significant effects of pesticide toxicity on taxa groups. We then used generalized linear models (GLMs) to assess the effects of pesticide toxicity, stream width and canopy cover on the total number of flying insects, the proportion of aquatic flying insects, the average number of prey detections per spider at each site, and the proportion of aquatic insects detected in the spider diet. Pesticide toxicity, stream width and canopy cover were first tested using spider sex as a response variable to ensure that the diets of male and female spiders could be analysed together. As spider body size can affect the size of prey that they are able to consume [64], we also checked whether there was an effect of body size (opisthosoma length) on the dietary composition of the spiders.

Finally, for each taxa group, we calculated the percent of occurrence (POO) in the spider diet and in Malaise traps at each stream site (i.e. the count of a taxa group divided by the total count of all groups for each site). We then divided the spider diet POO by the sum of the spider diet and Malaise trap POOs for each shared taxon group. The resulting ratio compares prey occurrence in the spider diet with the availability of that group in the sampled flying insect community. We then used GLMs to test the effects of pesticide toxicity, stream width and canopy cover on the occurrence ratios of each taxa group in the spider diet.

For all GLMs, we used the negative binomial family (‘glm.nb’, MASS; [65]) for count data, binomial family with permutation for proportional data and the Gaussian family with permutation for interval data. Model assumptions, including multicollinearity, were assessed using ‘check_model’ (performance; [66]) and ‘vif’ (car; [67]). All statistical analyses were performed using R (v. 4.2.2; [68]) and figures were created using ggplot2 and ggpubr [69,70]. A significant effect was considered when *p* < 0.05. Electronic supplementary material, table S11, presents the complete results of the statistical tests.

## 3. Results

### (a) Environmental gradients

**Figure 1.**
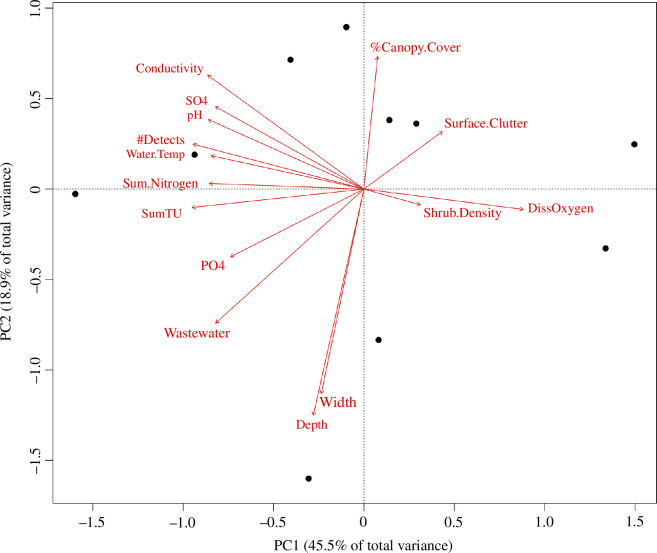
A principal component analysis (PCA) of environmental variables (red arrows) measured at stream sites (black points). Abbreviations: Depth: water depth, Width: stream width, Shrub.Density: separation score of shrubs along stream shore, DissOxygen: average concentration of dissolved oxygen, Surface.Clutter: average score of water surface coverage by vegetation clutter, %Canopy.cover: percentage of tree canopy cover, Conductivity: average water conductivity, SO4: average concentration of dissolved sulfate, pH: average pH of stream water, #Detects: average number of pesticides detected, Water.Temp: average water temperature, Sum.Nitrogen: average concentration of dissolved nitrate, nitrite and ammonium combined, SumTU: average sum of pesticide toxicity for freshwater invertebrates, PO4: average concentration of dissolved phosphate, Wastewater: total number of wastewater indicators detected.

The studied streams formed a gradient of chemical pollution. This was based on pesticide detections and toxicity, wastewater pollution, dissolved nutrients and other variables related to water quality across the stream sites, but also included a weak association with shrub separation (figure 1). The pesticide toxicity was highly correlated with almost all other stream pollution variables (electronic supplementary material, table S12, figure 1). The chemical pollution gradient aligned with the first PCA axis. The second gradient, consisting of stream width and depth, as well as canopy cover and vegetation characteristics (other than shrub separation), aligned with the second PCA axis. The pollution and stream size gradients were orthogonal and, thus, largely independent of each other.

### (b) Flying insect activity and prey shift

**Figure 2.**
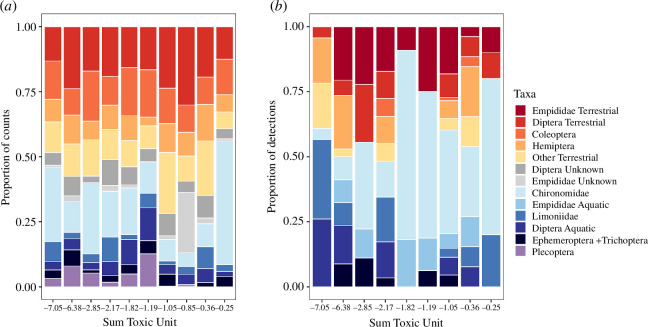
Composition of flying insects sampled with Malaise traps ((*a*) proportion of total insect counts) and the diet composition of *Tetragnatha montana* spiders ((*b*) proportion of prey detections from DNA metabarcoding) sampled at different stream sites in riparian forests. Stream sites are arranged from low to high chemical pollution and are labelled by their average pesticide toxicity (sum toxic unit) on the *x*-axis. A less negative sum toxic unit (log scale) signifies a higher toxicity, and a more negative value a lower toxicity. Taxa coloured from red to yellow (top) are terrestrial, blue and purple (bottom) are aquatic and grey (middle) are of mixed origin. Note that in (*a*), 25 individuals from aquatic families are included in ‘Coleoptera’, and that Empididae were not separated into aquatic and terrestrial groups (‘Empididae Unknown’). Site ‘−0.85’ is only included in (*a*) as no prey taxa were detected in spiders there.

The flying insect community at the streams was a mixture of terrestrial and aquatic taxa, with an average of 35 ± 13% of taxa identified as aquatic (figure 2*a*, electronic supplementary material, table S13). There tended to be fewer flying insects sampled at sites with higher pesticide toxicity (*t*_(6)_ = −2.52, *p* = 0.060) and canopy cover (*t*_(6)_ = −2.13, *p* = 0.061), but the proportion of aquatic insects trapped at each site did not have significant relationships with any of the tested environmental variables (*p* > 0.19; electronic supplementary material, table S11, figure S4). Diptera was the most numerous order of flying insects at all streams, with terrestrial Diptera (19.1 ± 1.7%) and Chironomidae (18.5 ± 4.0%) dominating the insects sampled by the Malaise traps both proportionally (figure 2*a*) and numerically (electronic supplementary material, figure S4). There was no significant overall relationship between the taxonomic composition of flying insects and in-stream pesticide toxicity (*F*_(1,9)_ = 1.56, *p* = 0.238), canopy cover (*F*_(1,9)_ = 2.25, *p* = 0.100) or stream size (*F*_(1,9)_ = 0.46, *p* = 0.707). However, there were significantly fewer Limoniidae (*t*_(8)_ = −2.13, *p* = 0.046) and especially few Plecoptera (*t*_(8)_ = −3.11, *p* = 0.017) at streams with a higher pesticide toxicity; only three of the 189 Plecoptera individuals were found at streams with a sumTU above −1.1, with none found at the two most polluted streams (figure 2*a*).

DNA metabarcoding of the collected spiders (electronic supplementary material, table S7) produced 4 785 262 total reads, of which 90.5% were identified as *T. montana* and excluded. The average number of reads per sample was approximately 22 500. After filtering, 150 258 reads were allocated to prey taxa, which clustered into 200 prey OTUs. Spiders consumed 105 taxa in total, 62 of which were aquatic. On average, 1.3 ± 2.0 taxa (maximum 14) were detected per spider, except for one site where we detected no prey DNA in any spiders so this was excluded from further analyses. There was no difference between male and female spider diet composition (*F*_(1,14)_ = 1.03, *p* = 0.397), number of prey detections (*t*_(14)_ = −1.09, *p* = 0.289), or proportion of aquatic prey (*t*_(14)_ = −1.64, *p* = 0.134), although females generally had slightly more prey detections (female average: 1.3 ± 0.3, male average: 0.8 ± 0.2) and slightly more aquatic prey (female average: 68 ± 5%, male average: 52 ± 8%) in their diet. There was also no significant difference in the diet composition of spiders of different body sizes (*F*_(1,8)_ = 0.663, *p* = 0.557).

The diet of *T. montana* comprised 64 ± 5% aquatic prey, on average (figure 2*b*). Diptera were the most common prey taxa group, with Chironomidae (35 ± 8%), Empididae (22 ± 4%) and Limoniidae (10 ± 3%) representing the most frequently consumed families. While the overall diet composition of *T. montana* did not differ significantly with stream size (*F*_(1,8)_ = 0.34, *p* = 0.793), pesticide toxicity (*F*_(1,8)_ = 3.10, *p* = 0.092) or canopy cover (*F*_(1,8)_ = 0.22, *p* = 0.896), spiders consumed significantly more Chironomidae (*t*_(7)_ = 2.54, *p* = 0.031) and fewer other aquatic Diptera (i.e. ‘Aquatic Diptera’: Culicidae, Dixidae, Dolichopodidae, Pediciidae, Psychodidae, Ptychopteridae, Simuliidae and Tipulidae) at more polluted sites (*t*_(7)_ = −3.20, *p* = 0.022; figure 2*b*). There were no significant relationships between stream pollution, canopy cover or stream size and the number of prey detections per spider (*p* > 0.21), the proportion of aquatic prey in the spider diet (*p* > 0.39), or the diet composition at the species level (*p* > 0.22; electronic supplementary material, table S11).

**Figure 3.**
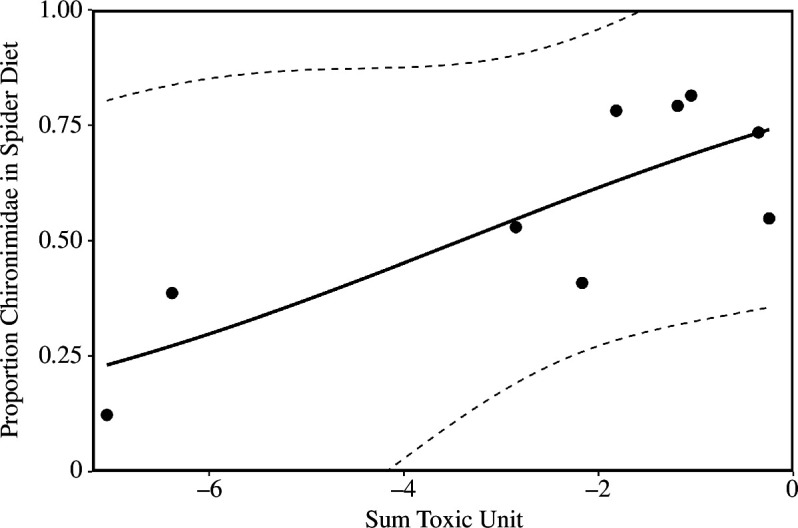
Relationship between the proportion of Chironimidae occurring in the diet of *Tetragnatha montana* spiders and the average pesticide mixture toxicity (sum toxic unit) measured at 10 forested streams (points). DNA metabarcoding was used to detect insect prey in spiders from the streams. Flying insects were collected at the streams with Malaise traps. The proportion represents the percent of occurrence (POO) of Chironimidae originating from the spider diet compared with the flying insects. A less negative sum toxic unit (log scale) signifies a higher toxicity, and a more negative value a lower toxicity. The dashed lines show the 95% confidence intervals.

The occurrence ratio of most insect taxa groups in the spider diet compared to in the flying insect community did not differ significantly among streams. However, the ratio of Chironomidae occurrence was higher in the spider diet at streams with a higher pesticide toxicity (*z* = 0.525, *p* = 0.010; figure 3, electronic supplementary material, table S14). In addition, the ratio of aquatic insects was lower in spiders than in the sampled flying insect community at streams with higher tree canopy cover (*z* = −0.336, *p* = 0.015; electronic supplementary material, figure S5).

## 4. Discussion

### (a) Effect of stream pollution on *Tetragnatha* diet

As expected, chemical pollution in streams was associated with a shift in the diet composition of *T. montana*, which consumed more chironomids and fewer other aquatic Diptera at more polluted sites (figure 2*b*). This finding was the strongest evidence for an indirect effect of stream pollution on *T. montana*, as we found no significant changes either in the number of prey detections or in the proportion of consumed aquatic prey. The effect we observed was likely a combination of chemical pollution together with other highly correlated variables such as increased water temperature and higher nutrient concentrations at polluted streams, as shown by the PCA (figure 1), which has been previously observed in similar streams (e.g. [71]). The results of our study expand upon those from a stable isotope-based study by Graf *et al*. [25], who found that the proportion of aquatic prey in the diet of *T. montana* along agricultural streams in Romania changed with intensive agricultural land use in the catchment but not with in-stream pesticide toxicity. Here, we show that a shift in the taxa consumed by spiders can occur in response to toxicity and stream pollution gradients, even when the overall proportion of aquatic prey in the diet does not appear to change.

Although the percentage of aquatic prey consumed by *T. montana* was high at all sites, chironomids clearly dominated the diet composition at sites with a high pollution load (sumTU > −2; figures 2*b* and 3). Potential explanations for this dietary shift may be an increased availability of chironomids owing to a change from sensitive to tolerant species emerging from the streams [71]. In another study on German streams, Liess *et al*. [62] observed a significant reduction in the proportion of sensitive aquatic invertebrates, as well as an increase in the abundance of tolerant taxa, at streams with TU > −3. Certain chironomids are well-known to be tolerant to pollution [33,72], which may explain their increased detection in the spider diet at more polluted streams in our study. Temporal differences in the peaks of chironomid emergence may also contribute to the differences between sites because although the spiders were sampled in similar time periods, emergence timing may shift at polluted streams [32].

However, the increased detection of chironomids in *T. montana* diet at polluted sites was not reflected by higher chironomid catches in Malaise traps. Apart from the possibility that Malaise trap catches are too stochastic to fully reflect differences in flying insect availability between streams, we can envision two possible reasons for this difference. First, the higher consumption of chironomids at polluted streams may suggest prey switching of *T. montana*. There are few studies describing the diet of *T. montana* other than in general terms such as ‘small aquatic dipterans’ [16,73], a diet that was confirmed by our metabarcoding data. Thus, *T. montana* may have preferentially consumed chironomids at polluted sites and switched to other prey at less polluted sites where more diverse taxa occurred. Second, it is possible that there is a mismatch between the sampling of flying insects using Malaise traps and spider web capture. *Tetragnatha* spiders are attracted to areas rich in aquatic prey [13] and they build their webs directly over the water surface. Our ‘SLAM’-style Malaise traps were located on the stream shore and 1 m above the water level. It may be that weak-flying chironomids were better ‘sampled’ by the spiders than the Malaise traps. Using additional methods such as benthos sampling, different trap positions and constructions, such as emergence traps (as in [32]), and further assessing the prey selection of *T. montana* (as in [74]) will help to more fully explain the differences between the flying insect community and spider diet compositions seen in our results.

### (b) Effect of stream pollution on available flying insect prey

There were no strong effects of chemical stream pollution on the overall flying insect community composition, although there were fewer Limoniidae and especially few Plecoptera at the most polluted streams. Plecoptera are highly sensitive to stream water temperature, habitat degradation and pollutants [33,72,75]. Similarly, many Limoniidae species are sensitive to pesticide pollution (based on SPEAR index, retrieved from freshwaterecology.info). Both Plecoptera and Limoniidae were probably absent from polluted streams owing to a combined effect of poorer water quality, less oxygen and higher water temperature, together with more dissolved nutrients and pollutants.

Chemical pollution from pesticides [62] and wastewater [76] can cause a shift in the macroinvertebrate stream community from diverse assemblages in terms of taxa, sensitivity and ecological traits, to more uniform communities. However, this shift may not always be detectable when measuring the flying insect abundance [31,77], and the effects of stream pollution observed in this study were not able to be disentangled from those of other drivers, such as temperature or stream productivity. Other factors that were not evaluated in this study, such as the presence of fish [78] or variation in sediment conditions [75], may also have influenced the flying aquatic insects emerging at streams, although we selected sites to be as similar as possible in both these regards.

### (c) Using DNA metabarcoding for analysis of dietary effects

Many studies evaluating the effects of contaminants on consumers of aquatic insects have used stable isotope analysis (SIA) to evaluate changes in their diets. While SIA is effective at determining the predator’s trophic level, the aquatic signature of the diet, the quantitative contribution of prey groups, and can reflect a longer temporal snapshot, it can also be misinterpreted and leave unanswered questions, particularly when the signatures of specific taxa are not easily distinguished [79]. Using DNA metabarcoding enabled us to detect a dietary shift in *T. montana* that had not been previously observed. We would probably not have seen an effect of stream pollution in our study using only SIA because there were no clear differences in the overall proportion of aquatic prey detected between streams.

Given this, we propose that DNA metabarcoding is an excellent method to complement the more quantitative results obtained with SIA and to examine dietary changes in greater detail, especially when SIA results are unclear, as shown by Hambäck *et al*. [30] and in Graf *et al*. [25]. Furthermore, knowing which species are consumed by spiders can reveal additional information about the prey community, such as general size composition, sensitivity to stressors or feeding traits. However, there are also limitations to DNA metabarcoding, such as lack of quantitative data in terms of individuals consumed [80] or sensitivity to contamination [28], which must be taken into consideration [26,29]. Although our goal here was not to compare diet analysis methods, we see that with the right considerations of its benefits and limitations, DNA metabarcoding has great potential for deepening our current knowledge of aquatic–terrestrial food webs and their responses to stressors.

### (d) Consequences of dietary shift

The shift of *T. montana* diet towards more chironomids at polluted sites while maintaining the proportion of consumed aquatic prey suggests an increased risk of pollutant uptake for spiders, rather than a dietary shift towards consuming more terrestrial prey. Increasing evidence shows that emergent insects can accumulate and export certain pesticides [81], metals [82] and pharmaceuticals from streams [83]. The presence of these compounds has also been associated with effects in consumers, such as changes in the microbiome of spiders [84] and bats [85] near wastewater treatment plants. Kraus *et al*. [86] summarizes possible consequences of in-stream pollutants for consumers of emergent insects: pollutants may act to reduce the availability of emergent insect prey (‘exposure driving subsidies’) and insect emergence may bioaccumulate certain pollutants, resulting in a pollutant transfer to consumers (‘subsidies driving exposure’). Although both dynamics may co-occur, as in the case of highly bioaccumulating and highly toxic compounds [87], the levels of stream pollution observed at our sites did not decrease the overall abundance of insect prey. Furthermore, Roodt *et al*. [88] found that certain pesticides accumulated and were biomagnified in riparian *Tetragnatha* spp. in our study area via emergent insects from the stream. Given this, we can expect that the spiders in our study were similarly exposed to contaminants at more polluted streams via their emergent insect prey. Pollutant exposure can result in negative consequences for spiders, such as possible poorer body condition due to sublethal effects [89], as well as pollutant transfer to the greater riparian food web. However, the effects of pesticide and wastewater pollutant exposure on spiders and riparian ecosystems remain largely unknown.

It should be noted that the effects we observed may differ situationally, as we only considered the spider diet during early summer at temperate streams. Insect emergence and its importance to riparian spiders vary across the seasons [12,32,71,90]. Thus, the transfer of contaminants to the riparian food web may also change seasonally. Conducting studies over a longer time frame would confirm whether the effects of pollution continue beyond the main pesticide application period. In addition, while the results of our study can be generalized to temperate streams with similar contamination profiles, the strength of subsidy- and exposure-driven effects seen here may change with different pollution levels, in different global regions, or in combination with different stressors. For example, arthropod predators in tropical regions were more reliant on emergent insects than in temperate regions [91], which may make them more susceptible to changes in emergence or to accumulating pesticides from streams. Investigating the effects of agricultural and urban pollution in streams from different areas would reveal whether the effects we observed are more widely applicable.

## 5. Conclusion

We found that *Tetragnatha montana* consumed more chironomids at more polluted streams but did not show a significant change in the overall proportion of aquatic prey in their diet. Their continued reliance on aquatic prey at polluted streams probably resulted in an increased dietary exposure of spiders to chemical pollutants, which could affect the spiders themselves and propagate further into the riparian ecosystem. In addition, the chemical pollution in the streams was probably one of several factors—including dissolved nutrients and temperature—affecting the insect emergence. DNA metabarcoding also proved highly suitable to detect shifts in the diet of *T. montana* along a gradient of stream pollution. Riparian spiders are one of the first links between emergent insects and the terrestrial ecosystems. Thus, changes to spider diet, or in the accumulation of contaminants, can affect their terrestrial predators and prey [17] and lead to further effects that alter the riparian ecosystem [16]. Chemical stream pollution occurs globally, and our results add to the evidence that its role as a stressor for biologically important riparian ecosystems should be taken seriously, especially as they face a multitude of stressors. We suggest that future studies investigate and identify the possible consequences of the observed dietary shift and potential exposure to chemical pollutants for riparian spiders and their food webs.

## Data Availability

The data for this study are available at Figshare [92]. Supplementary material is available online [93].
